# The Influence of Exercise on the Well‐being of Dementia Patients’ Caregivers

**DOI:** 10.1002/alz.093576

**Published:** 2025-01-09

**Authors:** Linda B Sewerbidges‐Williams

**Affiliations:** ^1^ Loma Linda University, Loma Linda, CA USA

## Abstract

**Background:**

The demands of caregiving can exact a toll on unpaid caregivers’ physical and emotional well‐being. Caregivers of individuals with dementia frequently experience high levels of stress, anxiety, and depression. Physical activity is a promising intervention for promoting the well‐being of dementia caregivers. Little is known about the association between one’s level of physical activity and the well‐being of unpaid dementia patients’ caregivers. The aim of the study was to investigate the association between physical activity and well‐being of unpaid caregivers of dementia patients.

**Methods:**

This cross‐sectional study included unpaid caregivers of dementia patients in Riverside, San Bernardino, Inyo, and Mono Counties, California. Utilizing a 31‐item questionnaire that included the International Physical Activity Questionnaire Short Form (IPAQ‐SF) and the Caregiver Self‐Assessment Questionnaire (CSAQ), participants provided insights into their physical activity levels and well‐being from July to October 2023. Logistic regression models were employed to analyze the relationship between physical activity (sedentary behavior, moderate physical activity, vigorous physical activity) and caregiver well‐being while controlling for demographic and health factors.

**Results:**

The survey included 152 mostly female (82.8%) and married (72.8%) respondents, with an average age of 56 years (SD = 8.4 years). The majority were aged 60 or older (65.6%) and had been caregivers for 3.5 years on average. More vigorous physical activity was associated with better well‐being (p < 0.05), but sedentary lifestyle and walking frequency was not. Gender and level of education influenced the association between physical activity and well‐being. Another notable aspect is that 85% of caregivers exhibited low well‐being.

**Conclusions:**

This study found an association between caregiver well‐being and vigorous physical activity but no association with sedentariness and frequency of walking. Designing personalized physical activity interventions maybe focusing on vigorous exercise can enhance the well‐being of dementia caregivers, thereby alleviating the burdens associated with dementia caregiving. Future research endeavors using longitudinal designs should be conducted to delve deeper into the underlying mechanisms driving these associations.

